# Growth hormone in disease and treatment (Review)

**DOI:** 10.3892/mi.2025.276

**Published:** 2025-10-22

**Authors:** Saikat Fakir, Md Matiur Rahman Sarker, Madan Sigdel, Nektarios Barabutis

**Affiliations:** School of Basic Pharmaceutical and Toxicological Sciences, College of Pharmacy, University of Louisiana Monroe, Monroe, LA 71201, USA

**Keywords:** endothelium, GHRH, somatostatin, inflammation, acute lung injury

## Abstract

Growth hormone (GH) is a peptide hormone produced by the anterior pituitary gland, which regulates growth and development. Abnormal levels of GH have been associated with a diverse variety of disorders affecting life quality and longevity; including dwarfism, acromegaly, gigantism and cancer. Based on the fact that growth hormone-releasing hormone (GHRH) and somatostatin exert opposing effects on the regulation of GH, GHRH antagonists (GHRHAnts) and synthetic somatostatin analogs (SSAs) have been developed to alleviate GH-related illness. The present study provides information on the multifaceted role of GH in human health and disease. Furthermore, it summarizes recent findings on the protective effects of GHRHAnts and FDA-approved SSAs, such as octreotide, lanreotide and pasireotide, in GH-related and endothelium-dependent dysfunctions. Based on the provided bibliography, an emerging body of evidence suggests that GH modulators may represent a promising therapeutic possibility towards blood brain barrier dysregulation, keratitis, direct and indirect lung injury, sepsis, and acute respiratory distress syndrome.

## 1. Endothelium

The endothelium lines the interior of blood vessels and functions as a barrier to maintain the movement of fluids, nutrients and immune cells between the bloodstream and surrounding tissues (1,2). It is involved in homeostasis, vascular contraction and cell growth regulation (3). Compromised endothelial function leads to vasoconstriction, thrombosis and increased permeability; which in turn contributes to the development of cardiovascular, metabolic and inflammatory lung disease (4,5).

In the lung, the endothelium maintains the blood-air barrier required for gas exchange and controls the vascular tone through the release of vasodilators and vasoconstrictors. Furthermore, it modulates immune responses by adhesion molecule and cytokine regulation. In the eye, the endothelium forms the blood-retinal barrier, and regulates fluid and solute transport between the aqueous humor and the corneal stroma. Damage to corneal endothelial cells may lead to impaired fluid regulation, corneal clouding, vision loss and Fuchs endothelial corneal dystrophy (6-9).

## 2. Endothelial dysfunction: A key player in vascular disease pathogenesis

Endothelial dysfunction impairs vascular homeostasis (10) and contributes in atherosclerosis, diabetic retinopathy, acute respiratory distress syndrome (ARDS) and multiple sclerosis (11-13). The abnormal expression of adhesion molecules (e.g., intercellular adhesion molecule 1, vascular cell adhesion molecule 1 and E-selectin) and pro-inflammatory mediators [e.g., tumor necrosis factor α (TNF-α), interleukin (IL)-1β and vascular endothelial growth factor (VEGF)] triggers actin-myosin contraction, the disruption of junctional proteins and paracellular gap formation. Leucocyte infiltration increases vascular permeability and results to plasma protein leak (14-16).

The dysfunctional endothelium becomes pro-thrombotic by releasing von Willebrand factor, which contributes to thrombosis and vascular disease (17,18). Under normal physiological conditions, endothelial nitric oxide (NO) synthase (eNOS) produces NO from L-arginine in the presence of tetrahydrobiopterin. NO is often decreased due to an increase in the levels of oxidative stress (19,20). This disruption in the electron transport process of the enzyme leads to the generation of superoxide (21,22). NO maintains vascular homeostasis, promotes vasodilation, inhibits platelet aggregation and reduces inflammation (23). Inflammatory cytokines (TNF-α and IL-1β) activate nuclear factor kappa-light-chain-enhancer of activated B cells (NF-κB) (24) and triggers inflammation, which in severe cases may lead to endothelial apoptosis or necrosis (25).

## 3. Inflammation

Inflammation is a complex biological mechanism which involves immune cells, blood vessels, and molecular mediators (e.g., microRNAs, adipokines and inflammasomes); to eliminate cell injury, remove necrotic cells, and initiate tissue repair (26). Acute inflammation is an immediate, adaptive response with limited specificity caused by noxious stimuli (e.g., infection and tissue damage) and protects against infection (27,28). Chronic inflammation has been associated with cancer, diabetes, neurodegenerative disease, pulmonary and autoimmune disease (29,30). It can potentially damage healthy cells, tissues and organs; since it promotes fibrosis, vascular damage, and immune dysregulation (31). Growth hormone (GH)-releasing hormone (GHRH), which regulates the release of GH from the anterior pituitary gland, promotes inflammation (32).

## 4. Pharmacological approaches to ameliorate endothelial dysfunction

Targeted pharmacological interventions are required to restore endothelial function in cardiovascular, renal, metabolic and respiratory disorders (33). Angiotensin-converting enzyme inhibitors, β blockers, dihydropyridine Ca^2+^ channel blockers, anti-oxidative agents (e.g., vitamins C and E, and N-acetylcysteine), phosphodiesterase inhibitors, statins, angiotensin, bradykinin and eNOS transcription enhancer exhibit strong potential to ameliorate endothelial dysfunction (24).

Emerging evidence suggests that growth hormone-releasing hormone antagonists (GHRHAnts), synthetic somatostatin analogues (SSAs) and never in mitosis A-related kinase 2 inhibition may enhance endothelial function in *in vitro* and *in vivo* models of lung endothelial injury (34-37). They can inhibit the the release of pro-inflammatory cytokines and counteract endothelial hyper-permeability (38). Furthermore, SSAs downregulate VEGF (39) and modulate tight junctions (40,41).

## 5. Growth hormone

GH, also known as somatotropin, is secreted by somatotropic cells which are located in the anterior lobe of the pituitary gland (42). This peptide hormone is involved in growth and metabolic regulation, affecting various physiological systems (e.g., neural, reproductive, immune, cardiovascular and pulmonary) (32). The GH level increases throughout childhood, reaching their peak during adolescence, stimulating the development of bone and cartilage (43). Following puberty, GH continues to maintain body composition, fat balance, muscle, bone tissue, insulin and blood sugar levels (44).

## 6. Physiological function of GH

GH secretion and release can be modulated by GHRH and somatostatin (SST) (45). GHRH stimulates GH secretion, whereas somatostatin exerts the opposing effects. GH receptors are ubiquitously present in the body. The binding of GH to its receptors initiates their dimerization, which occurs through the Janus kinase (JAK)/signal transducer and activator of transcription (STAT) pathway (46). The STAT proteins phosphorylate and translocate to the nucleus, triggering the transcription of target proteins [e.g., insulin-like growth factor-1 (IGF-1)]. IGF-1, produced in the liver, is a key mediator of the anabolic and mitogenic effects of GH (47).

GH stimulates lipolysis, protein synthesis, free fatty acid and glycerol levels, which contribute to decrease fat body mass (48). It also contributes to insulin resistance, particularly in type 1 diabetes (49). Furthermore, GH regulates fluid homeostasis by activating the renin-angiotensin system and stimulating distal renal tubular reabsorption, which leads to sodium and fluid retention (50). The multifaceted actions of GH highlight its indispensable role in health (51).

## 7. GH-related abnormalities

Abnormal GH levels lead to a variety of health issues (52). Excessive GH levels (acromegaly or gigantism) causes bone overgrowth in the hands, feet and face, along with other symptoms (e.g., joint pain and skin changes). Conversely, GH deficiency (GHD) can lead to fatigue, reduced muscle strength, increased body fat and decreased bone density (53).

### GHD

GHD is a condition characterized by inadequate secretion of GH from the anterior pituitary gland. In children, GHD manifests as short stature and growth failure, often accompanied by midface hypoplasia and increased truncal adiposity (54). Severe GHD may be apparent early in life with a significant reduction in height velocity (43) and can be congenital or acquired due to brain tumor, head trauma, or radiation therapy. Isolated GHD (IGHD) is linked to gene mutations [e.g., GH1 and GHRH receptor (GHRHR) gene] (55). IGHD type II is an autosomal dominant disorder, caused by GH1 mutations that affect the splicing of exon 3(56). This leads to the production of an abnormal GH (17.5 kDa) isoform, which disrupts hormone trafficking (57).

In adults, GHD results from structural pituitary or hypothalamic disorder or cranial irradiation (58). Adult-onset GHD is associated with a cluster of cardiovascular risk factors, including increased adiposity (particularly visceral fat), reduced muscle strength, impaired psychological well-being, insulin resistance, adverse lipid profiles and reduced bone mineral density (59). Patients may experience depression, difficulty concentrating, memory issues and anxiety or emotional distress. GH replacement therapy has been shown to reverse a number of these biological changes, improving body composition and overall health status (60).

### Gigantism

Gigantism is characterized by abnormally high linear growth due to excessive GH and IGF-1 levels prior to the epiphyseal growth plates fuse during childhood (61). In the majority of cases, gigantism is caused by a benign (non-cancerous) pituitary tumor, known as an adenoma, that hyper-secretes GH (62). Genetic mutations are also associated with the formation of pituitary tumors, leading to gigantism (63). Excessive hypothalamic GHRH levels may activate mutations in hypothalamic GHRH-neurons (64). The symptoms of gigantism include accelerated growth velocity, tall stature, enlarged hands and feet, soft-tissue thickening, prognathism (protruding jaw), coarse facial features, muscle weakness, cardiovascular issues, joint issues and headaches (65).

### Acromegaly

In the event that excessive GH secretion occurs following epiphyseal closure, the condition is referred to as acromegaly, which presents with similar features to gigantism but without increased linear growth. When the body produces excessive GH levels due to a non-cancerous tumor in the pituitary gland, it can lead to acromegaly (61). This hormone imbalance causes bones and tissues to grow to a greater extent than usual and has been associated with high blood pressure, diabetes and heart disease (66).

### GH and cancer

Excessive GH secretion increases the risk of developing colon, thyroid, gastric, breast, and urinary tract cancers (67). Specific genetic polymorphisms within the GH/IGF-1 pathway may increase susceptibility to malignancies (e.g., breast cancer) (68). Conversely, GH and IGF-1 deficits are associated with a diminished incidence of tumor promotion (69), associated to IGF-1/IGF-1R (70). IGF-1 promotes cell proliferation, differentiation and growth (71).

## 8. Growth hormone-releasing hormone antagonists

GHRHAnts were developed by Dr A.V. Schally (1926-2024) (Nobel Prize in Physiology or Medicine, 1977) to suppress malignancies (72,73); however, they have the potential to be used in a broader variety of disorders (74,75). These synthetic peptides reduce pituitary GH release and hepatic IGF-1 levels, and act directly on peripheral tissues by blocking both GHRHR and its splice variant SV1 (72,76-79). The activation of the cyclic adenosine monophosphate (cAMP)/protein kinase A (PKA) response element-binding protein signaling pathway leads to GH gene transcription and downstream IGF-1 production. GHRHAnts modulate key cellular processes involved in inflammation, oxidative stress, fibrosis and barrier integrity (80).

In peripheral tissues, GHRHAnts exert their protective effects by suppressing the activation of NF-κB (76) and inhibiting mitogen-activated protein kinases (MAPKs), such as ERK1/2 and p38 (81,82). Moreover, GHRHAnts suppress JAK/STAT signaling, particularly STAT3 activation, which is implicated in cytokine-driven inflammation and fibrosis (83). Those peptides can also mitigate ER stress through the induction of the unfolded protein response (UPR) (84), promote antioxidant defenses, and preserve barrier integrity (85).

The anti-inflammatory activities of GHRHAnts (74,82) have prompted their evaluation in experimental models of acute lung injury, pulmonary fibrosis and neuroinflammation (76,86). Moreover, GHRHAnts suppress pro-inflammatory cytokines, improve barrier function, and reduce oxidative and endoplasmic reticulum stress (82,87). Their ability to interfere with multiple cellular signaling pathways makes them particularly versatile for treating diseases (76,81,83).

### Experimental cancer therapy

GHRHAnts inhibit pituitary GH secretion and lower downstream IGF-1 secretion (78,88). By targeting GHRHR they can disrupt autocrine and paracrine pathways that promote cancer cell proliferation, survival, angiogenesis and metastasis (72,79,89). In preclinical experimental models, GHRHAnts, including MIA-602(90), MIA-690(91), JV-1-36(92) and JV-1-38(93), have exhibited significant inhibitory effects on the development of colorectal, lung, breast, prostate and pancreatic cancers. These effects are partially due to the suppression of c-Myc, cyclin D1 and IGF-1R (94). By activating caspases and modifying the expression of pro- and anti-apoptotic proteins, including Bax and Bcl-2, GHRHAnts promote apoptosis (95,96).

GHRHAnts prevent angiogenesis by reducing matrix metalloproteinases (MMPs; MMP-2 and MMP-9) (97) and VEGF, which are vital for tumor invasion and metastasis (98). Moreover, they exhibit minimal systemic toxicity and do not significantly suppress basal GH or IGF-1 levels at therapeutic doses (73). It has been suggested that GHRHAnts may serve as promising candidates for the treatment of cancer, either as monotherapy or in combination with chemotherapy, targeted agents, or immunotherapy, due to their capacity to target multiple tumor-promoting pathways (99).

### Acute lung injury (ALI) and ARDS

Barrier integrity is essential for maintaining homeostasis in the alveolar-capillary barrier (100), which is involved in the pathogenesis of complex disorders, such as ALI and ARDS (101). Those are severe and life-threatening pulmonary disorders characterized by uncontrolled pulmonary inflammation, increased vascular permeability, alveolar-capillary barrier disruption and impaired gas exchange (102). These conditions are frequently caused by infections, sepsis, trauma, or inhalation of toxic substances. Despite advances in supportive care, pharmacological options for ALI/ARDS remain limited. Recent research suggests that GHRHAnts protect against ALI and sepsis by simultaneously modulating key inflammatory processes and supporting the preservation of lung barrier integrity (103). MIA-602 has been demonstrated to exert protective effects in various ALI experimental models, including lipopolysaccharide (LPS)-induced endotoxemia and bleomycin-induced lung damage (104). GHRHAnts maintain vascular integrity by enhancing junctional stability and reducing pulmonary edema (105). Treatment with GHRHAnts has been shown to result in enhanced arterial oxygenation, lung injury amelioration and improved survival rates in animal experiments (103).

### Pulmonary fibrosis

Pulmonary fibrosis is a progressive life-threatening condition, which is characterized by the deposition of excessive extracellular matrix and alveolar architecture destruction, which result in loss of lung function (106). The chronic activation of fibroblasts and myofibroblasts is stimulated by profibrotic cytokines, such as transforming growth factor-β1 (TGF-β1) (107). Experimental evidence demonstrates that GHRHAnts exert anti-fibrotic effects (108). GHRHAnts (e.g., MIA-602 and MIA-690) have demonstrated a marked ability to inhibit key fibrotic processes by suppressing TGF-β1 signaling, preventing epithelial-mesenchymal transition, and decreasing fibroblast proliferation and differentiation into myofibroblasts (72,109,110). These peptides can also counteract the expression of profibrotic markers such as α-smooth muscle actin, fibronectin, type I collagen, and they can limit the deposition of collagen in lung tissue (76). In a previous study using a bleomycin-induced mouse model of lung fibrosis, GHRHAnts were shown to ameliorate fibrosis, improve lung compliance and preserve alveolar architecture (108).

### Blood-brain barrier (BBB) disruption and neuroinflammation

The BBB is a highly selective and specialized structure that regulates the exchange of molecules between the bloodstream and the central nervous system (111). The disruption of this barrier can lead to neuroinflammatory and neurodegenerative disorders, including multiple sclerosis, traumatic brain injury, ischemic stroke and Alzheimer's disease (112). The increased permeability of the BBB permits the entry of immune cells, cytokines and other harmful substances into the brain, triggering chronic inflammation, impairing neuronal function and exacerbating vascular damage (113).

There is experimental evidence to indicate that GHRHAnts can protect BBB integrity and reduce neuroinflammatory damage (114). In models of central nervous system injury, these peptides reduce the expression of inflammatory cytokines (e.g., IL-6 and TNF-α), suppress microglial and astrocyte activation, and block NF-κB (32). GHRHAnts stabilize the BBB and protect paracellular barrier integrity by maintaining tight junction protein (such as occludin and claudin-5) expression (115).

## 9. Somatostatin

SST, also known as GH-inhibiting hormone, exerts potent regulatory effects throughout the body (116). It functions as a neurotransmitter in the central nervous system (117), and exerts effects, at least partially, via the GH/IGF-1 axis (118). SST, which is affected by glucose, inhibits GH and thyroid-stimulating hormone secretion (119).

### Synthetic SST analogs (SSAs)

SSAs inhibit the production of GH and serotonin, which are used in managing GH-related disorders. Excessive GH secretion from pituitary adenomas leads to elevated levels of IGF-1(120). SSAs bind to SST receptors (SSTRs) on the surface of tumors in acromegaly and gigantism to inhibit the release of GH and reduce IGF-1 production (121).

### Octreotide

Octreotide is a Food and Drug Administration (FDA)-approved SSA (122). Due to its long half-life and high affinity for somatostatin receptor subtypes 2 and 5, it is widely used in clinical practice to manage disorders associated with excessive GH, such as acromegaly and gigantism (123). Octreotide suppresses the release of GH and reduces the hepatic production of IGF-1, leading to improved symptom control and biochemical normalization. Long-acting formulations of octreotide can normalize IGF-1 and GH levels (124), and shrink tumor volume (125).

Neuroendocrine tumors and GH-dependent cancers express SSTRs and respond to octreotide, which exerts anti-proliferative effects through receptor-mediated apoptosis, the inhibition of angiogenesis and the suppression of GH (126). It has been demonstrated that octreotide preserves endothelial barrier function, reduces reactive oxygen species (ROS) generation, and attenuates inflammatory responses in a murine model of LPS-induced ALI (34).

Octreotide may exert protective effects on vascular endothelial cells through the activation of the UPR and enhances the expression of ER chaperone proteins (e.g., binding immunoglobulin protein and glucose-regulated protein 94), leading to the stabilization of cytoskeletal components (127). When activating transcription factor 6 (ATF6) is pharmacologically inhibited, the beneficial effects of octreotide on endothelial permeability, ROS generation and improved cytoskeletal organization are virtually diminished (128). These findings suggest that octreotide confers protective effects on the vascular endothelium and may be useful towards inflammation-related vascular disorders.

### Lanreotide

Lanreotide is a long-acting SSA that exerts pharmacological effects by binding predominantly to SSTR2 and SSTR5(129), which are overexpressed in GH-secreting pituitary adenomas. Lanreotide has emerged as a valuable non-surgical therapy in acromegaly or in patients with residual disease following pituitary surgery (130) and may provide improved visual symptoms due to decreased tumor mass (131). The long half-life of lanreotide (23-30 days) and subcutaneous depot formulation offer convenient dosing, rendering it suitable for chronic conditions requiring long-term management (132).

Recent research suggests that lanreotide exerts protective effects against LPS-induced endothelial dysfunction both *in vitro* and *in vivo*. LPS typically induces endothelial hyperpermeability, leading to increased vascular leakage (133). Lanreotide reduces ROS generation in endothelial cells exposed to LPS, highlighting its role in mitigating oxidative stress. Furthermore, this FDA-approved analog ameliorated lung inflammatory disease (134).

### Pasireotide

Pasireotide, a second-generation SST analog, is distinguished by its high affinity for multiple SSRT subtypes (SSTR1-5) (135,136), and it is used in patients resistant to first-generation SSA (octreotide or lanreotide) (137,138). Pasireotide has been approved for use in patients with active acromegaly and Cushing's disease (139). Safety assessments reveal that pasireotide is generally well tolerated. The most frequently observed adverse event is hyperglycemia, a known effect of SSAs due to reduced insulin secretion; this can be managed with standard antidiabetic therapies. The tumor expression of SSTRs and dopamine receptors may predict the responsiveness to pasireotide in corticotropinomas (140).

Recent research suggests that pasireotide exerts potent protective effects on endothelial barrier integrity (35) and ALI, since it mitigates LPS-induced inflammation by preserving barrier function, reducing cytotoxicity, reinforcing antioxidant defenses, and dampening inflammatory responses. Mechanistically, pasireotide attenuates MAPK and JAK/STAT inflammatory signaling. These antioxidative and anti-inflammatory actions may synergistically stabilize endothelial junctions and suppress tissue injury (34,35,134,141).

### SSA administration

SSAs are available in both short-acting and long-acting formulations. Octreotide is available in subcutaneous and intramuscular forms (122), while lanreotide is delivered via deep subcutaneous injection (132). Pasireotide is administered either subcutaneously or as a long-acting depot injection (142,143) to modulate GH-related abnormalities (144).

## 10. Conclusions and future perspectives

**Figure 1:**
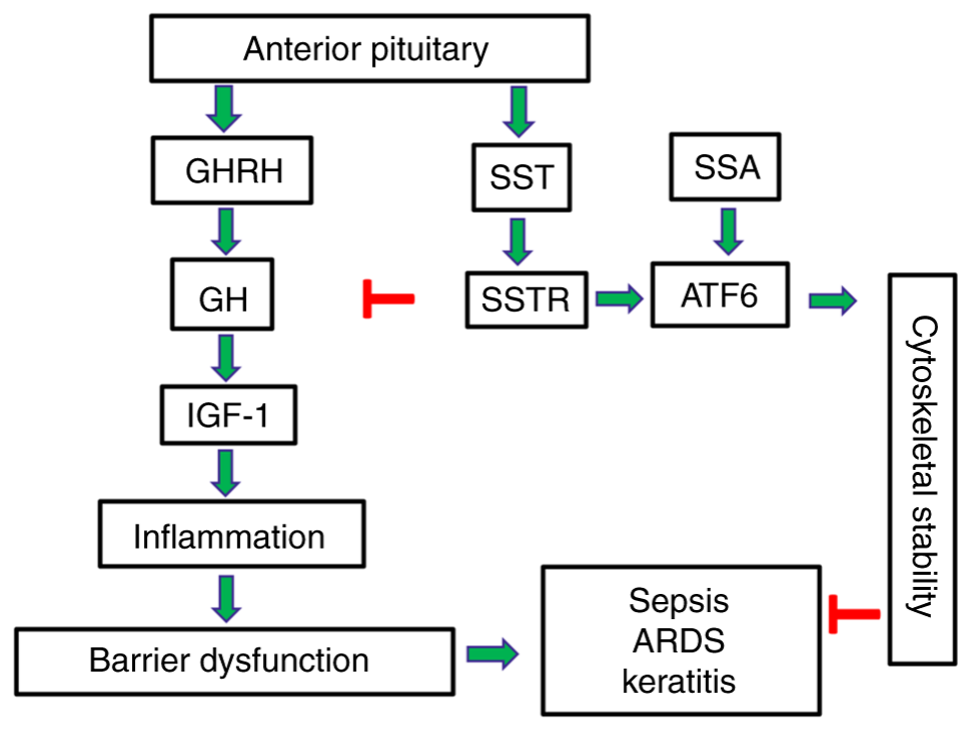
Involvement of GHRH and SST in endothelial barrier regulation and disease. GHRH regulates the secretion of GH from the anterior pituitary gland, which in turn induces IGF-1, inflammation and barrier dysfunction. SST counteracts GH-induced inflammation and activates ATF6 to support cytoskeletal integrity and ameliorate endothelium-dependent disorders (e.g. sepsis, ARDS and keratitis). GHRH, growth hormone-releasing hormone; SST, somatostatin; GH, growth hormone; IGF-1, insulin-like growth factor 1; ATF6, activating transcription factor 6; ARDS, acute respiratory distress syndrome; SSTR, somatostatin receptor; SSA, synthetic somatostatin analog.

Targeted GH modulation presents an exciting therapeutic intervention to alleviate human disease, and the development of synthetic analogs to achieve normal GH levels has been proven to be an effective strategy for the treatment of cancers and acromegaly in clinical practice (Table I). Recent preclinical studies suggest that SSAs and GHRHAnts counteract disorders related to endothelial barrier dysfunction utilizing ATF6 (127,128). Based on this information, experimental models of targeted ATF6 modulation will be developed to assist in designing GH-related pharmacotherapies. Those advanced approaches will be able to utilize the beneficial properties of UPR activation to strategically ameliorate endothelial-dependent disorders which may affect the brain, lungs and eyes (Fig. 1).

## Figures and Tables

**Table I:** FDA-approved GH-, GHRH- and SST-related analogs, their key effects and clinical applications.

Category	Name of analog	Effect	Application	(Refs.)
GH	Somatropin (recombinant human GH)	• Stimulates growth, protein synthesis and lipolysis • Increases IGF-1 production	• GH deficiency • Turner syndrome • Prader-Willi syndrome • Chronic renal insufficiency • Idiopathic short stature • SHOX deficiency	(43,47,144)
	Somatrem	• Promotes protein synthesis • Increases muscle cell size/number • Stimulates fat mobilization • Influences carbohydrate metabolism	• GH deficiency	(44,144)
GHRH	Tesamorelin	• Stimulates pituitary GH release • Increases IGF-1 and IGFBP-3 • Reduces abdominal fat accumulation	• HIV-associated lipodystrophy	(46,51,64)
GH receptor	Pegvisomant (antagonist)	• Blocks the GH receptor • Reduces IGF-1 levels	• Acromegaly	(46,143,144)
SST	Octreotide	• Inhibits GH, insulin, glucagon and GI hormones • Reduces splanchnic blood flow	• Acromegaly • Carcinoid tumors • VIPomas • Glucagonomas • Insulinomas • Thyrotropin-secreting adenomas • Esophageal variceal bleeding	(117,128,143)
	Lanreotide	• Suppression of cAMP • Modulation of K^+^ and Ca²^+^ currents • Inhibits signals that promote tumor growth or angiogenesis	• Acromegaly • Gastroenteropancreatic • Neuroendocrine tumors	(123,131,143)
	Pasireotide	• Broader somatostatin receptor binding • Stronger GH and ACTH inhibition	• Acromegaly resistant to other therapies • Cushing's disease	(129,137,143)

GH, growth hormone; GHRH, growth hormone-releasing hormone; SST, somatostatin; IGF-1, insulin-like growth factor 1; cAMP, cyclic adenosine monophosphate; ACTH, adrenocorticotropic hormone.

## Data Availability

Not applicable.
